# Application of agroforestry business models to tropical peatland restoration

**DOI:** 10.1007/s13280-021-01595-x

**Published:** 2021-07-06

**Authors:** Grahame Applegate, Blair Freeman, Benjamin Tular, Latifa Sitadevi, Timothy C. Jessup

**Affiliations:** 1 Tropical Forests and People Research Centre, University of the Sunshine Coast, Locked Bag 4, Maroochydore, 4557 Australia; 2 Indufor Asia Pacific, Level 8, 276 Flinders Street, Melbourne, 3000 Australia; 3 Global Green Growth Institute, Lippo Kuningan Building, 19th floor, Jl. Rasuna Said Kav. B12, Jakarta, 12920 Indonesia; 4 90 Sippy Downs Drive, Sippy Downs, QLD 4556 Australia

**Keywords:** Agroforestry, Indonesia, Investment, Restoration, Tropical peatlands

## Abstract

Indonesia is home to around 45% of the world’s tropical peatlands which continue to be degraded on a large scale by deforestation, drainage and fire, contributing massively to global GHG emissions. Approaches to restoring the peat–water balance and reducing emissions in peat hydrological units, through managing them based either on full protection or large-scale commercial production, have generally failed to address environmental and local community needs. We present published and unpublished findings pointing to the need for an integrated peatland protection and restoration strategy based first on raising water levels in degraded (drained) peatlands and maintaining them in forested peatlands, thus, reducing GHG emissions. Second, the strategy incorporates ecologically sound agroforestry business models that strengthen livelihoods of smallholders and so sustain their interest in sustainably managing the peatlands. In this paper, we focus on the second element of this strategy in Indonesia. Eight agroforestry business models are proposed based on their merits to attract both smallholders and commercial investors as well as their compatibility with hydrological rehabilitation of the peatlands. While financial returns on investment will vary across sites and countries, our analysis indicates that some models can be profitable over both short and longer time periods with relatively low levels of investment risk.

## Introduction

Tropical peatlands are estimated to cover an area of about 60 million ha (Yu et al. 2010; Page et al. 2011) and represent between 8 and 12% of all peatlands worldwide. A large proportion of these tropical peatlands are in South America (notably Peru), the Congo Basin, Malaysia and Indonesia (Dargie et al. 2017; Leifeld and Menichetti 2018). Based on current government figures and previous studies, it is estimated that Indonesia has between 30 and 45% of the world’s tropical peatlands (Harrison et al. 2020) representing between 7 and 11% of its land area (Osaki et al. 2016).

Large areas of peatlands have been subjected to deforestation, peat fires, drainage and conversion to agricultural and forestry plantations. The multiple ecological impacts of these disturbances have included dramatic changes to the hydrology causing large fluctuation in the water table of a metre in ‘peat domes’ (peatland hydrological units), as well as a broad range of other greenhouse gasses (GHG) and particulates (Stockwell et al. 2016; Jayarathne et al. 2018). Hooijer et al. (2010) established a linear relationship between the water table depth and CO_2_ emissions in degraded and drained peatlands in Kalimantan where the water table depths varied between 0.5 and 1.0 m. Under these conditions, emission can vary between 17 and 27 t CO_2_ ha^−1^ year^−1^ (Jauhiainen et al. 2005; Hirano et al. 2007) and up to 60 t CO_2_ ha^−1^ year^−1^ (Hooijer et al. 2012), with 9 t CO_2_ ha^−1^ year^−1^ released per 10 cm of drainage as reported by Couwenberg et al. (2010). Restoring the water table level and rewetting the peat through canal blocking in a large part of a peat hydrological unit to close to original levels could avoid up to 60–70 t CO_2_ ha^−1^ year^−1^ (van Ruiten and Suyanto 2009), while concurrently reducing the loss of biodiversity through maintaining water levels in surrounding indigenous peat swamp forests (PSF) and the conversion of peatlands from a carbon sink to a source of CO_2_ (Hooijer et al. 2010; Joosten 2010; Frolking et al. 2011; Leifeld 2013; IPCC 2014).

In Indonesia, large-scale peat fires have been observed in degraded peatland during severe dry periods since 1997 (Page et al. 2002), contributing also to increases in GHGs through this oxidation process. Extensive fires in 2015 burnt approximately 2.6 million ha and caused an estimated 16 USD thousand million in damages to natural resources, agricultural production and infrastructure (Glauber and Gunawan 2016).

Ongoing land-use change and disturbance across Indonesia’s peatlands contribute approximately 60% of Indonesia’s overall GHG emissions (Peatland Restoration Agency 2016). Reducing the hydrological degradation and restoring these peatlands by “rewetting”—specifically, blocking and infilling the canals and, thus, raising water table levels and protecting the remaining natural vegetation cover often found in the central and deeper part of the peat dome—can significantly reduce GHG emissions (Dommain et al. 2010; Dohong et al. 2017; Jessup et al. 2020), with reduced incidence of peat fires and protection of restored and remaining PSF (Giessen 2015; Applegate 2017).

Peatland restoration in Indonesia has progressed to varying stages of planning and implementation across the country. None of the current efforts cover the entire peatland biome, and most site-specific initiatives do not cover entire hydrological units. This limits their effectiveness in managing water flow and maintaining high water tables to keep the peat moist and, thus, reduce GHG emissions and fire impacts in the long term. Nevertheless, it is clear that a key element of success is the participation and support of local stakeholders—particularly smallholder farmers and fishers with legitimate and secure rights to resources in and around the peatlands—and that solutions to the problems of peatland restoration and protection must offer tangible economic benefits to those stakeholders. Hence, we argue that an integrated peatland protection, restoration and management strategy is required, which incorporates agroforestry businesses as elements necessary to provide for sustainable livelihoods, which in turn will assist to mainstream landscape-scale adoption of restorative and sustainable practices (Smith 2014). Thus, while the agroforestry elements by themselves are insufficient in the absence of canal blocking and other interventions to rewet and restore degraded peatlands, they must be compatible with those interventions and they must provide sustainable economic benefits.

## Integrated peatland protection, restoration and management strategy

It is now widely recognized that many tropical hydrological peatland units or peat domes consist of a number of peat land types which can be categorized into land-use zones as a basis for restoration and protection. In recognition of these features, we along with others have promoted the development of a strategy for the sustainable management of tropical peatlands in Indonesia, based on an integrated approach to peatland management and restoration, across entire peat hydrological units (Peatland Restoration Agency 2016; Applegate et al. 2018; Jessup et al. 2020). An integrated approach to peatland management, including land-use zonation, across the whole of the hydrological unit allows for the whole-of-dome approach to water management aimed at rewetting drained areas by blocking the canals. Rewetting has been found by a number of researchers to be the most effective means of reducing GHGs both from fire and biological decomposition (Page et al. 2009; Jacnicke et al. 2011; Peatland Restoration Agency 2016). Maintaining water levels across all or most of the dome allows for limited access to the deep peat to conduct restoration works and promote the development of PSF tree species (Wichtmann et al. 2016; GGGI 2018a); and use of the shallow peat and surrounding mineral soils for agriculture and forestry for local income generation. The specific types of activity and zonation should be based on a participatory approach with the biophysical and social legitimacy formalized in local development plans (Jessup et al. 2020). The biophysical zoning will need to consider hydrological conditions (such as water table depth), peat depth and degree of degradation, as defined by the terms used for the peat types as follows:

- *Non-peat* the term given to mineral soils, but can include “peaty” soils which contain an organically rich upper layer of less than 50 cm.
- *Shallow peat* the term given to peat which is shallow-to-medium in depth (i.e. 50 cm to 3 m) forming a buffer around the deep peat. Shallow peat is defined in legislation in Indonesia as peat that is less than 3 m deep. However, for agricultural purposes, rural communities grow crops in ‘shallow peat’ that is often between 0 and 60 to 200 cm deep and where the water table is usually 60 cm below the surface for much of the year. In some areas of shallow peat, rewetting and sensitive treatment of the peat maybe necessary. These conditions are usually found close to river systems and on bunds, which often comprise peat and mineral soil formed during the excavation of the canals.
- *Deep peat* the term given to peat that is > 3 m deep which is not degraded, or which can be rewetted and protected.

**Fig. 1:**
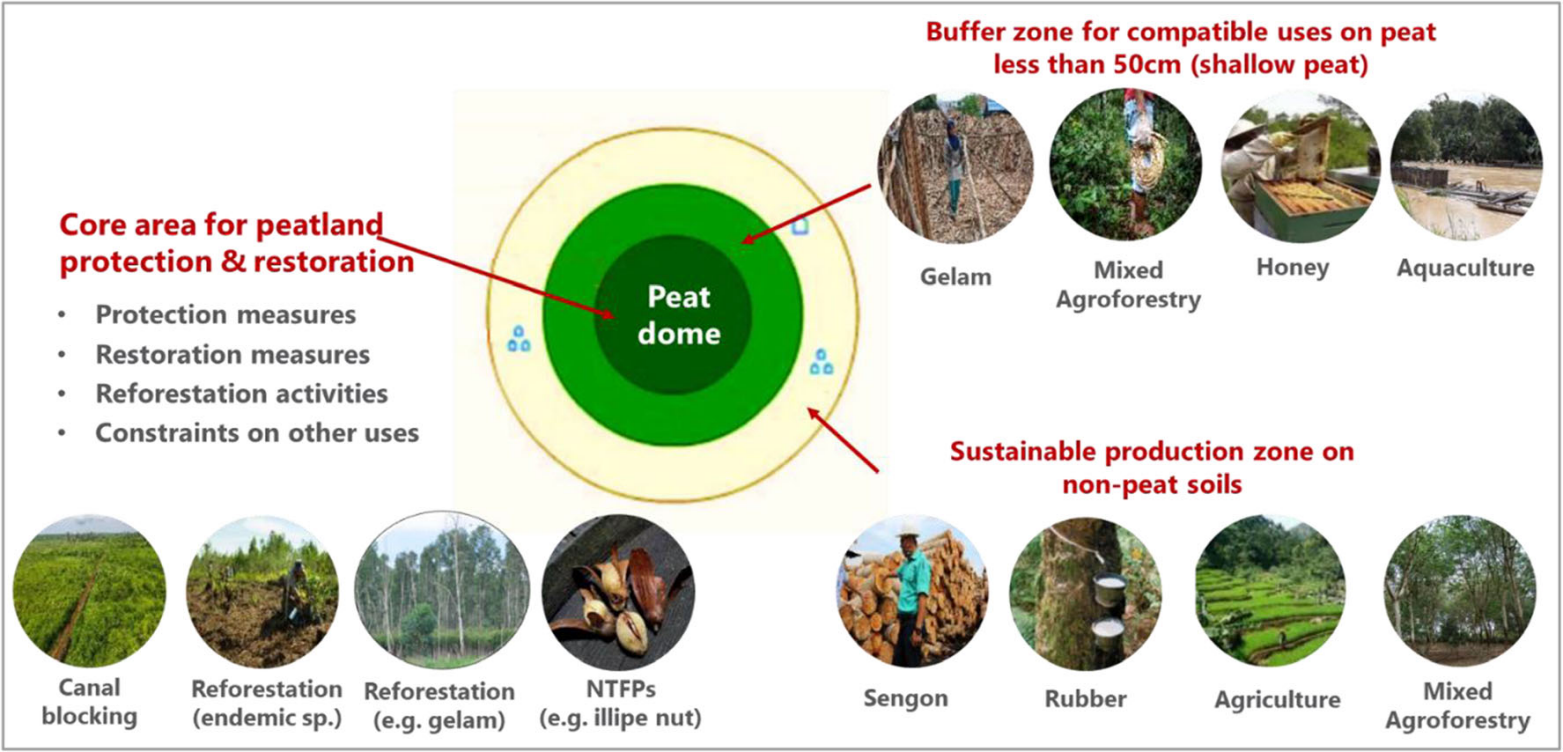
Peatland zoning identifying suitable land-use options for an integrated approach to peatland protection and restoration

This zonation of peatlands and appropriate land uses, including agroforestry, are shown in a schematic of the integrated peatland restoration strategy (Fig. 1); originally outlined in Applegate et al. (2018) and Jessup et al. (2020) (Table 1).

**Table 1:** Suite of eight agroforestry business models for a range of peat categories that complement the integrated peatland restoration strategy

Peatland profile	Peatland agroforestry models	
Mineral soils (surrounding peatlands)	Model 1	Sengon (*Falcataria moluccana*) and cash crops on mineral soils
Shallow peat	Model 2	Sengon (*Falcataria moluccana*) and cash crops on shallow peat bunds
	Model 3	Gelam (*Melaleuca leucadendra*) and honey production
	Model 4	Coffee (*Coffea liberica*)-based agroforestry systems
	Model 5	Fish ponds and productive (timber, fruit) trees
	Model 6	Sago (*Metroxylon sagu*) based agroforestry systems
Deep, rewetted peat	Model 7	Native Peat Swamp Forest trees, paludiculture and honey
	Model 8	Gelam (*Melaleuca leucadendra*) and honey on rewetted peat

Improving community-based livelihood options based on agroforestry businesses on non-peat soils and shallow peat areas in the surrounding buffer zones will assist in securing community support for protection and restoration within the deeper peat areas (i.e. canal blocking for rewetting the peat and reforestation). Successful agroforestry businesses will in turn contribute to the conservation of PSF, by providing alternative incomes to individuals or communities, improved land productivity and access to non-timber forest products such as honey and rattan. These are positive benefits for communities living within and around these areas (Osaki et al. 2016; Ministry of Environment and Forestry 2017; Research Development and Innovation Agency 2017).

To secure the integrity of the core deep peat zone for peat restoration and protection, a substantial buffer zone of sustainable land-based economic activities is required. This buffer zone may include agroforestry as indicated in Fig. 1. These activities must be compatible with peat restoration, or rewetting of the deep peat zone and serve to redirect more intensive land uses away from the core zone, to protect it from drainage, the use of fire and other destructive land-use practices that will lead to increased GHG emissions and further loss of biodiversity. The buffer zone, comprising shallow peat and non-peat (mineral) soils or peaty soils, can provide for community-based activities and other enterprises that generate income and livelihood security for farmers and a reasonable return on commercial investment.

In addition, there is some scope for low-impact production on deep peat soils that will not impact adversely of the rewetting activities or on their natural state. These include growing and selective harvesting of products from PSF species (paludiculture) to support sustainable livelihoods for communities living on or adjacent to deep peat areas (Giessen 2015; Applegate et al. 2018).

## Agroforestry business models for peatland Restoration

The primary purpose of our study^1^ was to identify viable and sustainable agroforestry business models, as key elements of the integrated peatland protection and restoration and management strategy in Indonesia. The agroforestry models themselves do not directly lead to the permanent saturation of the peat by water and the restoration of the peat hydrology, or a reduction in GHGs (Page et al. 2009; Evers et al. 2017); that is the function of rewetting the peat through the canal blocking and fire prevention elements of the integrated strategy. However, the development of suitable agroforestry models can be directed towards strengthening the primary rewetting initiatives, to underpin carbon benefits. The agroforestry business models developed for the mineral soils, located around the edges of the peat hydrological unit are not subject to canal blocking to raise ground water table levels.

We undertook a synthesis of published research, along with an appraisal of grey literature and from our own field-based research relating to agricultural crops and tree species and the range of growing conditions on tropical peatlands. This synthesis was undertaken recognizing that effective agroforestry businesses need to be undertaken by local communities.

The species identified as having potential for agroforestry businesses are influenced by key parameters such as yield levels over time, the potential for revenue generation, land requirements relevant to revenue generation and scalability to be attractive for investment, as well as reduce GHG emissions and the risk of major fires (Graham 2009; Indonesia Australia Forest Carbon Partnership 2009; Sofiyuddin et al. 2012, 2013; Giessen 2015; Osaki et al. 2016; Perdana et al. 2016; Applegate 2017; Research Development and Innovation Agency 2017). We also incorporated results from our own primary field investigations of options for smallholder production of stakes, poles and timber from naturally grown *Melaleuca leucadendra* (gelam) (GGGI 2018a) and sawlog and peeler logs from plantation grown *Falcataria moluccana* (sengon) (GGGI 2018b).

**Fig. 2:**
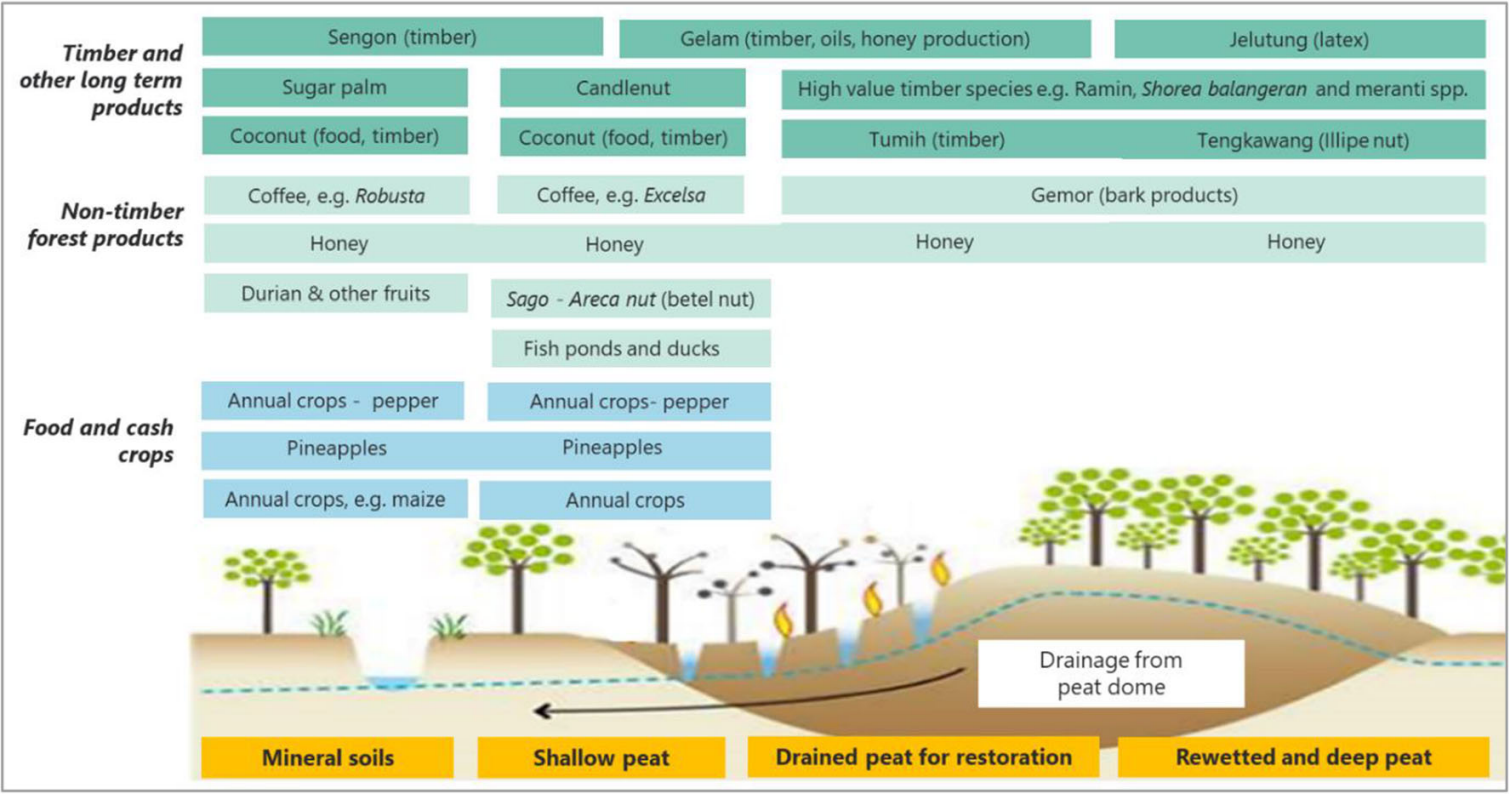
Summary of selected species

We prepared a non-exclusive list of species that could be planted effectively across the range of peat-zoning categories and have the potential for production in an agroforestry context for a range of products including food crops, timber and non-timber forest products for short- and longer-term income streams, including but not limited to rattan, honey, essential oils and latex products as shown in Fig. 2. The tree species listed in the ‘drained deep peat’ and ‘rewetted peat areas’ are all indigenous PSF species and are among the most suitable for paludiculture and agroforestry as well as for restoration and biodiversity conservation in the deep peat or protected zone.

Our study also revealed that in comparison to the literature on individual agricultural crops and tree-based paludiculture species, there is limited information on the performance of agroforestry systems established on peatland (Sofiyuddin et al. 2012; World Agroforestry Centre 2016; Japan International Cooperation Agency, JICA 2017). Similarly, there are relatively few studies that compare the financial returns from agricultural crops grown on tropical peat (Giessen 2015; JICA 2017).

In recognition that the integrated peatland management and restoration strategy involves land-use zoning of entire peatland hydrological units, we have proposed agroforestry business models that typically feature two or more primary species or tree species-based activities. The primary species were chosen based either on ecological or cash-crop value attributes, but they could be substituted in the models with other ‘primary’ species with similar attributes. They are ‘primary’ in the sense that their attributes have a dominant influence on the characteristics of the model or on the choice of suitable models for specific situations. However, these models are not limited to only those species; as in most cases, agroforestry models should provide scope for a range of complementary species. In this context, the primary species represent the anchor points for the model, with scope for variations on these themes.

Recognizing that single species are unlikely to be the best restoration solution, either for meeting the livelihood needs of communities or for re-establishing a well-functioning peatland landscape, we are proposing a suite of eight agroforestry business models (Table 1) with demonstrably sound commodity supply chains, which together with positive market values, are likely to attract and secure investment by local communities and the private sector. These models are applicable for peatlands in Indonesia as well as other tropical peatlands with similarities to those of Indonesia. Each of the models requires a set of investment pre-conditions and other key considerations, reflecting the individual factors for each site, including the biophysical and hydrological conditions, current land uses, community aspirations, market access and demand for agroforestry products that need to be addressed.

**Fig. 3:**
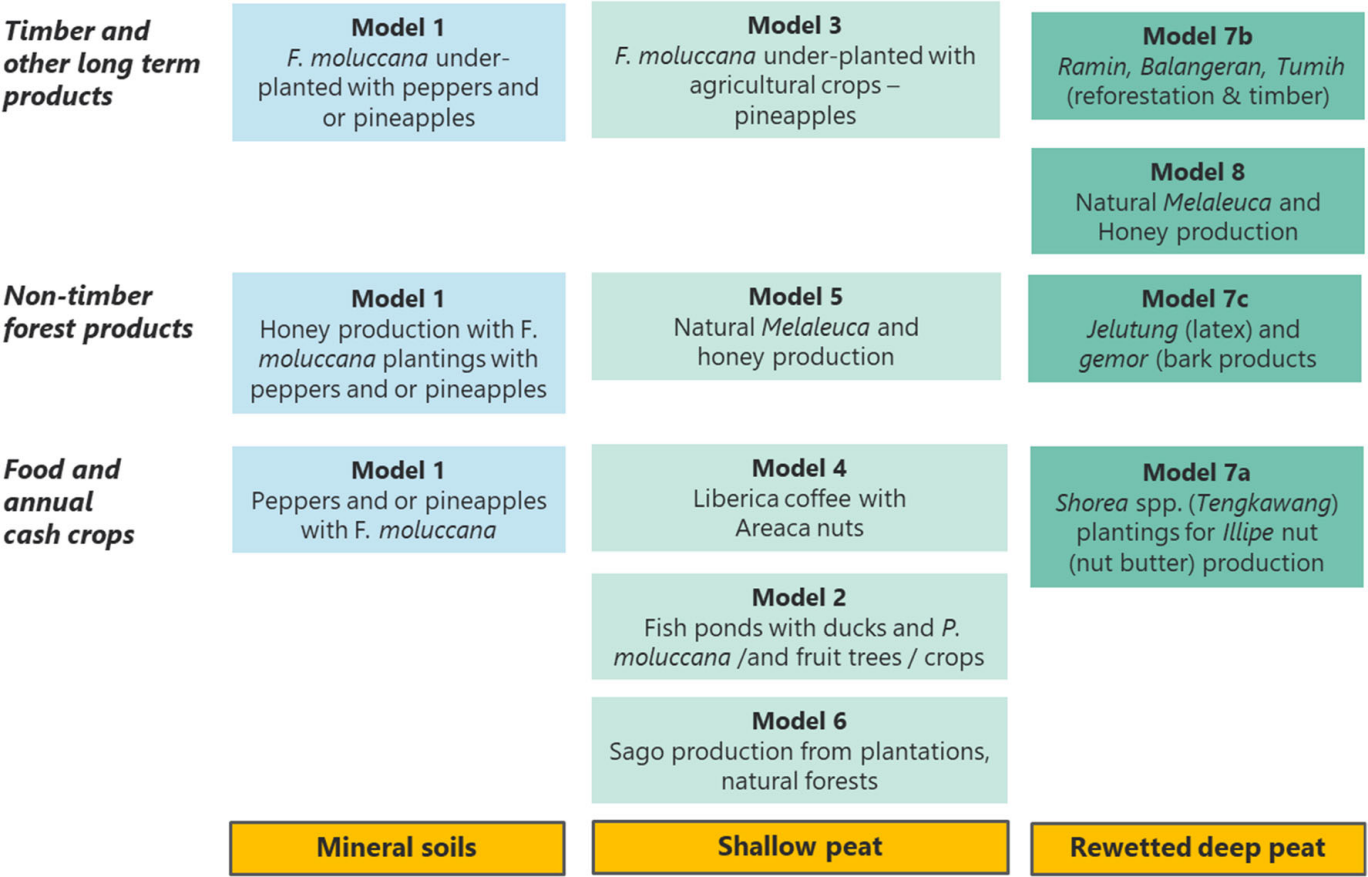
Summary of eight agroforestry business models

While several of the models are structurally distinct, others represent variations on a theme, adapted for different sites within peat landscapes. A schematic summary of these agroforestry business models showing their zone location on the peat dome is set out in Fig. 3.

The proposed models are described below. These descriptions contain references to published statements and financial returns derived from basic analyses, often made using a set of specific assumptions at a point in time. Costs and revenues would vary significantly across sites and the following data are presented for illustrative purposes only.

### Agroforestry models for mineral soils

#### Model 1: Sengon (*Falcataria moluccana*) planted together with cash crops

*Falcataria moluccana* is a fast-growing hardwood tree species that occurs naturally in Indonesia. It is a popular species, due to its many useful timber properties, its relatively high productivity across a range of soils and its nitrogen-fixing properties (GGGI 2018b). Growing sengon in an agroforestry system could provide income for individuals and local communities from the sale of logs after 8^2^ years and from the sale of agricultural crops growing beneath the sengon canopy, as well as from thinning of sengon stems at age 3–4 years and income at age 8 years from the final harvest.

Based on our study of the species and markets in parts of Indonesia (GGGI 2018b), we are proposing a model based on dividing the area available for agroforestry into uniform blocks; one for each year of the anticipated rotation length and numbered, i.e. 1 to 8. In year 1, Block 1 would be planted with sengon at a wide spacing (indicatively 4 m × 4 m or 3 m × 3 m), depending on the requirements for the inter-planted agricultural product and as part of the silvicultural management to promote good tree form. When the sengon is planted, the inter-row areas would also be established with two closely spaced rows of agricultural cash crops such as peppers or pineapples, and tended accordingly. The tending of the agricultural crop assists with the weed control for the sengon and reduces the risk of fire.

During year 1, the agricultural crops from Block 1 would be harvested, providing income to the grower. This system is then repeated until all blocks are planted. By age 3 or 4 years, Block 1 would be large enough to be thinned, thus, providing an additional income from thinning material. Hence, after the first rotation of approximately 8 years, income would be derived from the sale of sengon trees thinned in one block, one block fully harvested and two blocks with agricultural crop production. We analysed the returns from this model and found a smallholder with 2 ha of land available for sengon on a rotation of 8 years, with approximately half a hectare of peppers or pineapples inter-planted among the sengon and harvested annually, could produce an annual revenue of approximately 8000 USD for the peppers/pineapples and 5000 USD for the sengon (at the farm gate) in 2018. If honey production from apiculture was also included with the sengon trees, which provides shade for the hives and flowers for the honey production, the sengon would be able to sustain at least five hives, which could net up to an estimated 80 USD year.

### Agroforestry models for shallow peat

#### Model 2: Sengon (*Falcataria moluccana*) and cash crops

Model 1 can be adapted for application to shallow peat areas, which include the bunds on the edges of the canals comprised largely of mineral soil. Sengon can be established in blocks or rows and underplanted with agricultural cash crops, and this agroforestry model would generally be suitable both prior to and following rewetting of the deep peat zone. The bunds are generally elevated and, as a result, are well above the water table for much of the year and will remain so even when the deeper peat is restored by rewetting with canal blocking.

Our experience and field observations suggest that the production of sengon on these sites is likely to be less than it would be on mineral soils, but the potential for production of pineapples, for example (which are adapted to grow well on acid soils), as an underplanted crop in an agroforestry system will be enhanced by the nitrogen-fixing sengon. Therefore, the returns on sengon will generally be less than in Model 1, but the pineapples (if these are selected as the preferred cash crop) could have a gross revenue in the order of 2000 USD ha^−1^ if planted on half of the total area available. Although this is a general model which precludes definitive numbers on revenues and costs, it is a generic agroforestry system that can be applied across a broad range of sites, where suitable cash crops could include peanuts, chillies, eggplant and ginger.

#### Model 3: Gelam (*Melaleuca leucadendra*) on shallow peat

Gelam (*M. leucadendra*) grown on shallow peat produces two closely linked commodities: sawn timber and/or poles and honey (GGGI 2018a). Areas where gelam can be managed sustainably on shallow peat, or on deep peat inside protected zones, are highly compatible with honey production, and this is an enterprise that can involve the local community with minimal time inputs. Our study of gelam-harvesting enterprises in Indonesia suggests sustainable management, and harvesting of gelam can provide profits in the order of 10–15 USD ha^−1^ for a small group of cutters over a 6-month production period. The gelam is sold as stakes, poles and logs for sawn-timber production. Pole processors and saw-millers process and market these products, making gross incomes of around 50 USD m^−3^ and 130–200 USD m^−3^ for poles and sawn timber, respectively (GGGI 2018a).

Honey production (wild honey and/or apiculture) is highly complementary with the sustainable management and harvesting of gelam. There are two types of bees involved. The bees *Apis melifera* and *Apis cerena* produce honey from apiculture, while *Apis dorsata* produces wild honey. The two production systems are viable in the tropics and suitable for rehabilitated peatlands with forest species such as gelam and other indigenous PSF species required for conservation of biodiversity and for timber and non-timber products. Both honey production systems require large patches of forest which produce flowers all year round, which makes gelam forests highly suitable as it regenerates prolifically in wet peatlands and those exposed to flooding and fire.

Beekeeping has relatively low establishment costs (approximately 100 USD hive^−1^) and 60 USD hive^−1^ for annual maintenance and requires only a small area of land to locate the hives with minimal manual inputs. The gelam forests on the rehabilitated peatland provide a sustainable resource of pollen, a source of water and a suitable environment for the hives. The number of hives can vary between 15 and 20 hives per household and with bees foraging in forests over about 30 km^−2^, large areas of forests with good-quality pollen such as from gelam can support the production of 11–20 kg of honey per season per hive. Our research indicates that this type of agroforestry system can provide substantive returns on the honey alone of up to 200 USD to households per season per hive, which would enable local communities to recover their investment within 2–5 years.

#### Model 4: Coffee (*Coffea liberica*) based agroforestry

In some parts of Indonesia’s shallow peat area, lowland coffee (*C. liberica*) can be produced at scale (Sofiyuddin et al. 2013; JICA 2017). This is compatible with an agroforestry system incorporating *Areca* spp*.* (edible nuts) as a cover crop and bee keeping which takes advantage of the shade for the hives and flowers for honey production. At present, not only the demand for this type of coffee is relatively small in local markets, but it is also sold in Singapore and Malaysia at a higher price than either Robusta or Arabica coffee.

Areca nuts (*Areca catechu*) are often grown in an agroforestry system with the coffee. The nuts fetch a high price in Indian and Malaysian markets (JICA 2017). The price depends on the quality and type of nuts, and the post-harvest drying process. In 2017, Indonesian farmers were paid between 0.30 and 0.50 USD kg^−1^ for fresh nuts, while the price paid for dried nuts was just above 1 USD kg^−1^ (JICA 2017).

#### Model 5: Fishponds and productive trees

One of the most identifiable agroforestry systems on shallow peat in Indonesia is fishponds (traditional fisheries known as “beje”) in combination with the farming of ducks, trees for honey, fruit and timber, which are established on the bunds surrounding the ponds. This system relies on fluctuations in the movement of water or the overflow of river water during the rainy season when flood waters, along with small fish, enter the ponds via pipes and are trapped. The fish can breed in the ponds and develop to a marketable size and are typically harvested during the dry season when the water recedes. There are native ducks (*Anas platurynchos*) from southern Kalimantan, Indonesia (Sasongko and Guntoro 2012) that are a useful and profitable addition to the ponds. Our study showed that commercial tree species planted on the bunds not only provide shade and protection for the fish and ducks but also fruit or timber products when they mature. In some areas, sengon is a useful addition on the bunds if they are mainly composed of mineral soil. Our field observations and interviews indicate that fishponds of approximately 1000 m^−2^ require an investment of indicatively 15 000 USD, with annual incomes expected of around 2000 USD. As an example of the typical scale, bunds surrounding a fishpond with an area of 1000 m^−2^ could support at least 100 sengon trees planted two rows apart. Over a rotation length of 8 years, the sengon would produce indicatively 15–20 m^−3^ of merchantable sawlog or veneer log and be sold for around 500 USD. Raising 100 ducks in the ponds and duck eggs could provide an additional net annual income of around 1500 USD year^−1^ (JICA 2017).

#### Model 6: Sago (*Metroxylon sagu*) agroforestry systems

Sago (*M. sagu*) is a highly valued crop that represents another prospective core species when grown in plantations or managed as natural-assisted regeneration under different peatland conditions. Sago is best suited to coastal regions where there is tidal movement of water or where water fluctuations are provided artificially via canals, which also cause drainage and, thus, subsidence of the peat. Sago can grow well on shallow peat areas but is not a suitable species for deep peat areas, particularly deep peat areas requiring rehabilitation (Flach and Schuiling 1988).

Smallholders that produce plantation sago often interplant with perennial crops such as bananas and pineapples. However, most of the land suitable for sago is composed of peat soil that is acidic and is often subject to flooding, making it difficult to plant other crops. Plantations of sago have been successfully established on peatlands with the assistance of drainage canals to control water table depth and water flow in Sumatra (JICA 2017). Sago is planted as seedlings into prepared planting holes in cleared and drained peatland. The ‘clusters’ or shoots are produced and can be harvested every 10 years, with new ‘clusters’ forming on the original plant. Plantation development costs are in the order of 1000 USD ha^−1^.

Sago also grows well naturally on peat where it is mixed with mineral soil on the banks of river systems (Wardis 2014). In West Papua, we have observed natural stands of sago growing on shallow mineral soils over-topping deep peat. These stands are managed by thinning out unwanted vegetation and exposing the young sago palms. The sago grown in these areas produces less starch and takes longer to mature (more than 12–17 years) compared to 8–12 years when cultivated on shallow peats and mineral soils (JICA 2017). The poor growth of sago palms on deep peat is likely caused by the lack of nutrients in the peat strata rather than a low pH value. A single trunk of sago palm can produce between 150 and250 kg of dry sago flour, which was sold wholesale in 2017 in Indonesia for 2–3 USD kg^−1^ with processed sago flour retailing for 6 USD kg^−1^ (JICA 2017).

### Agroforestry models for deep peat

#### Model 7: Tree species (paludiculture)

This model comprising paludiculture using indigenous PSF species for conservation and the production of timber and honey is well suited to the deep peat areas. The deep peat is usually located in the centre of the peatland hydrological unit and by regulatory definition in Indonesia, typically exceed a depth of 3 m. When this is the case, no cultivation or cropping should be permitted in this core area and it should be designated for protection and restoration through canal blocking to raise or maintain water table levels above 40 cm or maintained as natural peat swamp forest and managed accordingly.

There are variations of this agroforestry model that involve PSF species for biodiversity conservation and production of timber and non-timber products that are readily adapted to growing on deep peat areas with elevated water levels. We describe this model in three parts as follows:

- PSF species which produce timber and non-timber products as part of a paludiculture system (raised water levels) in association with honey production undertaken by villagers living close to the edge of peatland hydrological units (Graham 2009; Graham et al. 2016). Paludiculture can be a key component of the integrated peatland restoration model and has been promoted as part of the focus for the rehabilitation of the deep peat by the Indonesian Research Development and Innovation Agency (2017) and Jessup et al. (2020), to establish and manage a suite of species in different combinations. These Indonesian studies have identified over 30 PSF tree species suitable for paludiculture.
- PSF species such as *Shorea* spp. can be managed for conservation or planted to produce edible nuts for consumption and converting into ‘nut butter’. These ‘illipe nut’ species include *Shorea pinanga*, *S. macrocarpa*, *S. stenoptera* and *S. macrophylla* (JICA 2017). It is important to note that these species are not necessarily suitable for timber production but are suitable for conservation and restoration efforts in the deep peat as the nuts can be harvested without felling the trees.
- Appropriate, moderately fast-growing PSF species planted in mixtures suitable for producing high-quality timber (in the long term) and honey production grown in the rehabilitated peatland following canal blocking. Suitable species include Balangeran (*Shorea balangeran*), Tarantang (*Campnosperma coriaceum*), Geranggang (*Cratoxylon arborescens*), *Eugenia oblata*, *Alstonia spathulate* and *Calophyllum sclerophyllum* (ramin), *Gonystylus bancanus* and *Combretocarpus rotundatus* (Graham 2009). While these species can produce timber which has a commercial value, they are also indigenous PSF species, which provide fundamental building blocks for restoring the forests as they are tolerant of elevated water levels required for peatland restoration. These species can be planted from seedlings at a stocking of between 1100 and 1200 stems ha^−1^ (Graham et al. 2016; Research Development and Innovation Agency 2017). Sawn timber prices for these species in Indonesia range from 400 to 500 USD m^−3^ in 2018. On this basis, limited timber could be produced in the future when the trees are large and plans are developed to sustainably harvest and transport the logs using the *kuda kuda* system, involving narrow gauge railway systems laid out in the forest to remove the logs, thus, avoiding the use of destructive canals to transport the logs from the forest (Ibbotson 2014; JICA 2017).

Mixtures of PSF species can also be planted for biodiversity and for a broader range of non-timber products which can be produced without felling the trees, including Pantung/Jelutung (*Dyera polyphylla*) whose latex is used to manufacture chewing gum; and gemor (*Alseodaphne coriacea*) whose bark is used to manufacture insect repellent (Graham 2009; GRM International 2010; Graham et al. 2016; Perdana et al. 2016; Tata et al. 2016; Research Development and Innovation Agency 2017)*.*

#### Model 8: *Gelam* (*Melaleuca leucadendra*) on deep peat

This model is based partly on our field observations and on our interviews with smallholders and processors. It involves the integrated production of wooden stakes, poles and timber, together with honey production from natural stands of gelam in the deep peat zone. It is closely related to Model 3, with the key difference being its focus on the deep peat zone rather than the shallow peat zone, where the roots can often penetrate the mineral soils in search of additional nutrients. Gelam has the capacity to regenerate under increased and prolonged high water levels from coppice, stems and roots and while the trees cannot survive permanent inundation in rewetted the deep peat, it is fibrous, adventitious roots located at the base of the stem are ‘breathing roots’ to assist the tree to survive long periods when the base of the stem is underwater during the wet season. In the absence of fire, careful management with frequent thinning of stems to produce stakes and poles along with periodic patch felling of the larger stems will create the necessary disturbance to ensure regeneration from seed to develop a sustainable resource for the production of timber and non-timber forest products. Gelam has co-benefits for the honey industry as gelam flowers at different times throughout the year, enabling honey production through apiculture and collection of wild honey.

## Comparative assessment of the agroforestry business models

**Fig. 4:**
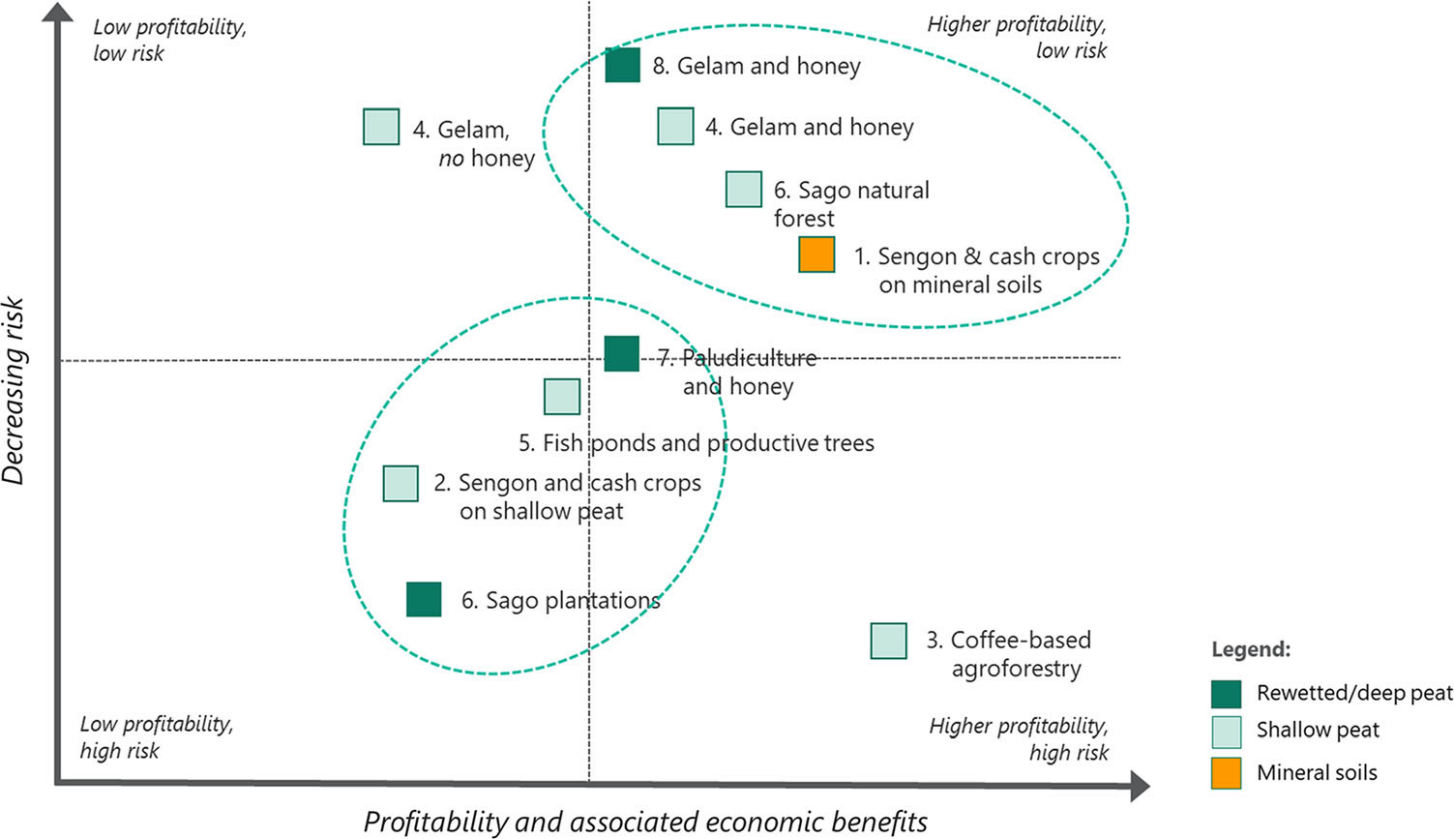
Comparative assessment of agroforestry models based on their indicative level of profitability and associated risks

**Fig. 5:**
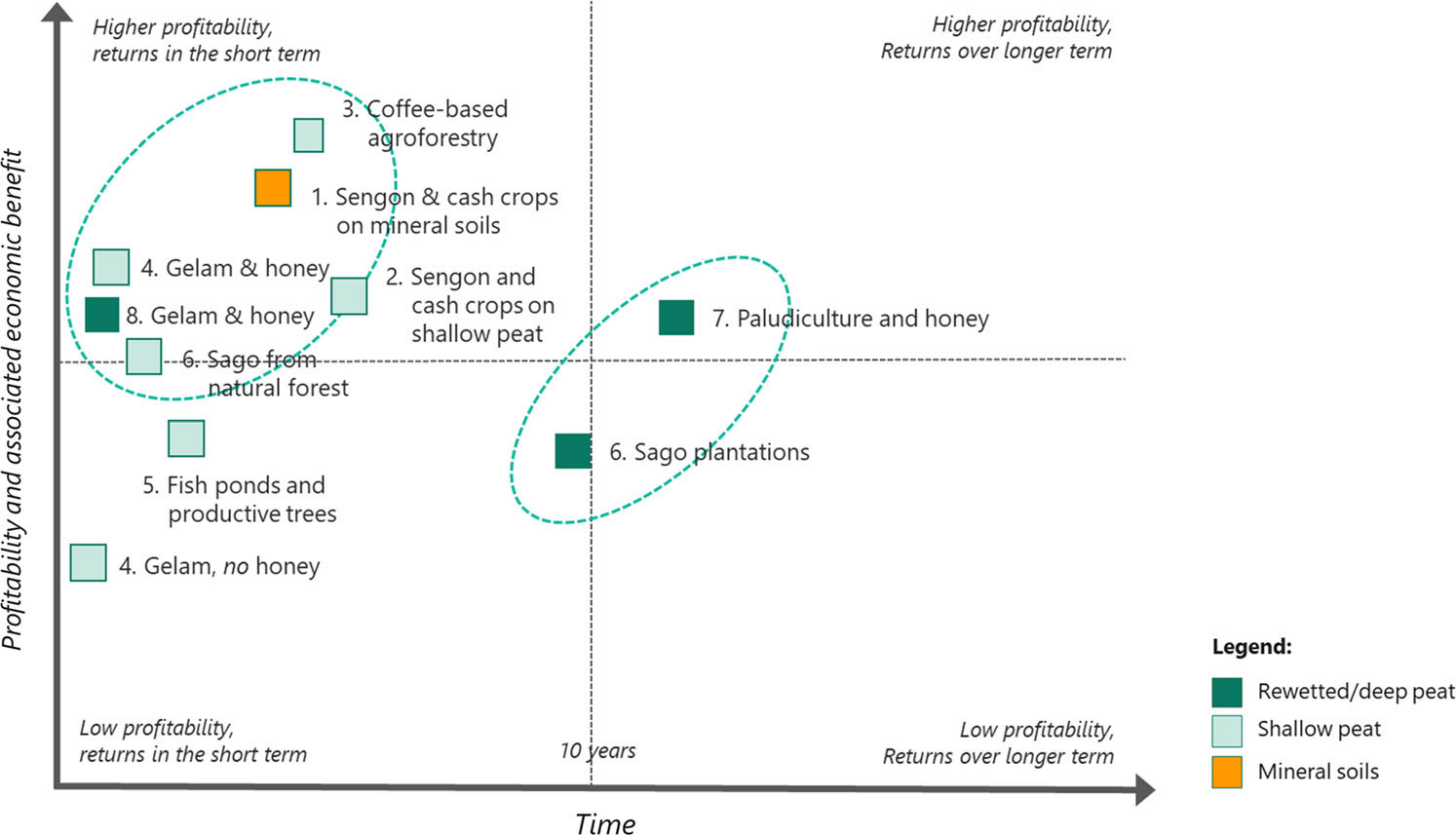
Comparative assessment of agroforestry models based on indicative timeframe for profitability and associated economic benefits

The options outlined above can be used by policymakers, prospective proponents and investors as base models, based on sound technical advice and recognizing that the parameters can be changed, including external price fluctuations, to suit specific locations or meet specific investment. The agroforestry models are presented schematically showing relative degrees of profitability and risk in Fig. 4 and relative profitability over time in Fig. 5.

Figure 4 highlights distinct clusters of the proposed agroforestry options. The first and leading cluster can be characterized as having the potential for higher profitability and lower risk. These options include the sengon plantations grown with cash crops on mineral soils, gelam on peatlands and sago-based agroforestry.

The second cluster can be characterized as options that tend to carry higher risk, for reasons that include limited information available on production and market requirements, as well as less favourable growing conditions (e.g. growing on peatlands). These options also tend to have lower profitability expectations based on commodity production alone, although the value of other ecosystem services—some of which can be monetized—may be higher.

The outlier option in this schematic is coffee-based agroforestry. This option is considered to have potentially high profitability, while also having a high level of risk, due mainly to climatic and soil factors relating to growing coffee on tropical lowland peatlands.

Figure 5 illustrates the relative profitability and the anticipated time horizon for realizing profitability from each agroforestry enterprise. Similarly, there are two main clusters. The first and leading cluster tends to have higher profitability expectations, with the potential to realize these net profits in a relatively short timeframe of between 3 and 8 years. There is considerable overlap in the identification of these leading options. They include the combination of sengon and cash crops, gelam wood and honey, and sago-based agroforestry in natural forests. In addition, the coffee-based agroforestry becomes part of the leading cluster due to its profitability in a relatively short period of time. The second cluster will take longer to realize a profit, in the order of 10 years and longer. These enterprises, comprising paludiculture with honey and sago plantations, typically require less time inputs (i.e. lower intensity management for communities) and may be preferred on this basis or for other factors relating to the specific site conditions.

## Conclusions

This paper has presented a comparative assessment of agroforestry business models that would be suitable for peatland restoration projects in Indonesia, through integrated strategies that focus on protecting the core peat zone and rewetting programmes, by developing sustainable production enterprises outside this zone, on surrounding shallow peat lands and mineral soils. The primary outcomes for these projects will typically be restoring the peat hydrology and reducing peatland degradation and loss of GHG emissions; and the primary mechanisms for achieving these outcomes will be rewetting the peat through the canal blocking and fire prevention elements of the integrated strategy.

The establishment of agroforestry models forms a secondary component of this integrated strategy. While the agroforestry models themselves do not lead directly to the permanent saturation of the peat by water and the restoration of the peat hydrology, they can strengthen the model by providing production enterprises and economic benefits for local communities, which will effectively secure the perimeter and reduce the risk of further incursions into the core peat zone.

The range of agroforestry business model options that may be suitable are subject to site-specific conditions and management objectives. The preferred options for specific sites will depend on various factors, encompassing hydrological conditions, current land use and tenure, peat characteristics, the importance of conservation or protection and proximity to markets. An ‘options-by-context’ approach should be adopted, whereby areas designated for productive functions are developed for agroforestry, paludiculture or other environmentally compatible land management practices, while other areas designated for protection are restored based on a peat swamp forest ecosystem model. This approach has been adopted by Indonesia’s Peatland Restoration Agency (BRG), according to current policies that divide peatlands (other than conservation areas) into ‘protection zones’ and ‘cultivation zones’. (Peatland Restoration Agency 2018).

It is unlikely that any single species or commodity will be the best solution for peatland restoration or protection, given the ecological, economic and cultural diversity of their landscapes. Our synthesis of published studies and grey literature on agroforestry models in Indonesia indicates that improved cash flow and profitability can be realized if appropriate combinations of commodities and tree-based systems are adopted as part of strategies for restoring, conserving and developing whole peatland hydrological units.

## Footnotes

1 The study was commissioned by the Global Green Growth Institute to shape the design of suitable business models for peatland rehabilitation. This study forms part of a larger initiative that aims to demonstrate that green investments are possible and can become the norm both for investors and for planners, ultimately improving the overall quality of economic growth and development.

2 This rotation length is based on medium to high productivity sites for sengon, and on poor soils, the rotation length would likely need to be longer.
